# CircMEMO1 modulates the promoter methylation and expression of TCF21 to regulate hepatocellular carcinoma progression and sorafenib treatment sensitivity

**DOI:** 10.1186/s12943-021-01361-3

**Published:** 2021-05-13

**Authors:** Zhao-Ru Dong, Ai-Wu Ke, Tao Li, Jia-Bing Cai, Ya-fei Yang, Wei Zhou, Guo-Ming Shi, Jia Fan

**Affiliations:** 1 Liver Cancer Institute, Zhongshan Hospital, Fudan University, 136 YiXue Yuan Road, Shanghai, 200032 China; 2 Key Laboratory of Carcinogenesis and Cancer Invasion (Fudan University), Ministry of Education, Shanghai, 200032 China; 3 Department of General Surgery, Qilu Hospital, Shandong University, Jinan, 250012 China; 4 Cancer Center, Institutes of Biomedical Sciences, Fudan University, Shanghai, 200031 China

**Keywords:** HCC, Circ RNA, miR-106b-5p, TET, TCF21

## Abstract

**Background:**

Cirrhosis is a recognized risk factor for developing hepatocellular carcinoma (HCC). Few studies have reported the expression profile of circRNAs in HCC samples compared to paratumour dysplastic nodule (DN) samples.

**Methods:**

The Arraystar Human circRNA Array combined with laser capture microdissection (LCM) was used to analyse the expression profile of circRNAs in HCC samples compared to paratumour DN samples. Then, both in vitro and in vivo HCC models were used to determine the role and mechanism of key circRNA in HCC progression and treatment sensitivity.

**Results:**

We found that circMEMO1 was significantly downregulated in HCC samples and that the level of circMEMO1 was closely related to the OS and disease-free survival (DFS) of HCC patients. Mechanistic analysis revealed that circMEMO1 can modulate the promoter methylation and gene expression of TCF21 to regulate HCC progression by acting as a sponge for miR-106b-5p, which targets the TET family of genes and increases the 5hmC level. More importantly, circMEMO1 can increase the sensitivity of HCC cells to sorafenib treatment.

**Conclusion:**

Our study determined that circMEMO1 can promote the demethylation and expression of TCF21 and can be considered a crucial epigenetic modifier in HCC progression.

**Supplementary Information:**

The online version contains supplementary material available at 10.1186/s12943-021-01361-3.

## Background

Hepatocellular carcinoma (HCC) is one of the most common malignancies and the third-most common cause of cancer-related death worldwide [1, 2]. The overall prognosis of HCC patients remains unsatisfactory, even after surgical treatment [3, 4]. Metastasis remains the most challenging problem that influences the prognosis of patients with HCC [5]. Despite recent significant progress in molecular targeted therapy and immunotherapy for HCC, sorafenib is still the first-line standard of treatment for many patients with advanced HCC, including patients with locally advanced HCC. Identification of key candidates that regulate the metastasis process and sensitivity to sorafenib treatment in HCC may contribute to improving patient prognosis and treatment effects.

The development of HCC in the cirrhotic liver is described as a multistep process that progresses from dysplastic nodules (DNs) to HCC foci, followed by small HCC and finally overt carcinoma. Circular RNAs (circRNAs), novel endogenous noncoding RNAs formed with a covalently closed loop, play crucial roles in tumorigenesis and tumour progression [6, 7]. Few studies have reported the expression profile of circRNAs in HCC samples compared to paratumour DN samples.

Transcription factor 21 (TCF21), a member of the class II bHLH transcription factor superfamily, has been demonstrated to be aberrantly methylated and frequently silenced in human malignancies [8–12]. TCF21 not only mediates cell fate and differentiation by orchestrating temporal and spatial gene expression during the development of various organs but is also recognized as a key regulator involved in a wide spectrum of essential biological processes, such as cell proliferation, differentiation, survival, cell cycle, invasion and metastasis [8, 10]. Owing to its crucial role in transcriptional regulation, TCF21 has great potential as an efficient therapeutic target for a number of cancers [12]. However, the regulatory mechanisms, including the reason for aberrant TCF21 dysregulation in HCC tissues, are still unsolved.

Here, the Arraystar Human circRNA Array combined with Laser Capture Microdissection (LCM) was used to identify that circMEMO1 was significantly downregulated in HCC tissue samples. The level of circMEMO1 in HCC tissues was closely related to the prognosis of HCC patients. Mechanistic analysis revealed that circMEMO1 can promote the demethylation process of the TCF21 promoter and then further activate the transcription and expression of TCF21 by acting as a sponge for miR-106b-5p, which targets TET family genes and increases the 5hmC level. In addition, circMEMO1 can increase the sensitivity of HCC cells to sorafenib treatment. Therefore, circMEMO1 can be considered a crucial epigenetic modifier to regulate HCC progression.

## Materials and methods

### Clinical specimens and patient follow-up

In total, 209 specimens were randomly collected from consecutive patients with HCC who underwent curative resection at the Liver Cancer Institute of Fudan University (Shanghai, China). Fresh human HCC and adjacent nontumour liver tissue samples were blindly collected from the cohort. Informed consent was obtained from each patient, and ethical approval was granted by the Ethics Committee of Zhongshan Hospital, Fudan University. The follow-up procedures were described in detail in our previous studies [2, 3, 13].

### Cell culture, transfection, and RNA extraction

The human HCC cell lines Huh-7, PLC/PRF/5, Hep3B, MHCC97H, and HCCLM3 were routinely maintained in our laboratory. Transient transfection was performed using Lipofectamine 2000 (Invitrogen), and total RNA was obtained using the mirVana™ miRNA Isolation Kit (Life Technologies) according to the manufacturer’s instructions.

### The Arraystar human circRNA Array analysis

Total RNA from each sample was quantified using a NanoDrop ND-1000. The sample preparation and microarray hybridization were performed based on Arraystar’s standard protocols. Briefly, total RNA from each sample was amplified and transcribed into fluorescent cRNA utilizing random primers according to Arraystar’s Super RNA Labeling protocol (Arraystar Inc.). The labelled cRNAs were hybridized onto the Arraystar Human circRNA Array. After washing the slides, the arrays were scanned by an Axon GenePix 4000B microarray scanner.

Scanned images were then imported into GenePix Pro 6.0 software (Axon) for grid alignment and data extraction. Quantile normalization and subsequent data processing were performed using the R software package. Differentially expressed circRNAs with statistical significance between two groups were identified through volcano plot and fold change filtering. Hierarchical clustering was performed to show the distinguishable circRNA expression pattern among samples.

### Plasmid construction

The lentiviral vectors **GV689** containing human circMEMO1 were constructed from Shanghai GeneChem, China. The shRNA targeting circMEMO1 or random sequence were synthesized and inserted into lentivirial vector **GV493** (**hU6-MCS-CBh-gcGFP-IRES-puromycin**) from Shanghai GeneChem, China. The lentiviral vectors pGMLV-SC5-shmiR-106b and pGMLV-MA2- pri-miR-106b and negative sequences were purchased from Gene Meditech (China). Human HCC cells were then transduced with the appropriate lentivirus. The plasmid pGMLV-SC5/pGMLV-MA2 expressed eGFP, and fluorescence was visualized to estimate the overall transfection efficiency. To select stably transduced cells, samples were resuspended and cultured with puromycin (2 μg/mL) for 2 weeks; quantitative reverse transcription-polymerase chain reaction (qRT-PCR) was then performed to determine the level of miR-106b-5p.

cDNA templates of ten-eleven translocation (TET) 1 were cloned into the pPB-CAG vector and transfected into HCC cells using Lipofectamine 2000 (Invitrogen) according to the manufacturer’s instructions. Stably transfected clones were selected and validated by qRT-PCR and immunoblotting.

### Quantitative real-time polymerase chain reaction (qRT-PCR), Western blot analysis, dot blot analysis, immunohistochemistry (IHC) and immunofluorescence assay

QRT-PCR, western blot analysis, dot blot analysis, IHC and immunofluorescence assays were performed as described previously [13, 14]. The primers for miR-106-5p during qRT-PCR were purchased from GeneCopoeia, Rockville, Maryland. The other PCR primer sequences were provided in Table S1. For 5-mC and 5hmC staining, samples were placed in 2 N HCl for 30 min, rinsed in distilled water, and placed in 100 mM Tris-HCl (pH 8.5) for 10 min before being blocked with PBS containing 5% BSA.

### Migration and invasion assays, in vivo metastasis assays

Migration and invasion assays and in vivo metastasis assays were performed as described previously [3, 13].

### Luciferase assay

The 3′-untranslated region (UTR) of human TET1/2 was amplified by PCR and cloned into the pGL3 vector to generate the pGL3-TET1/2–3’UTR. This construct (2 ng) was cotransfected with 2 ng of pRL-TK plasmid. Luciferase activity was measured and normalized 48 h after transfection. Alternatively, cells were cotransfected with 200 ng of luciferase plasmids, 2 ng of pRL-TK and 20 nM miRNA or miRNA inhibitor.

### In situ hybridization

Tissue microarray (TMA) slides or 4-μm-thick formalin-fixed paraffin-embedded (FFPE) samples were incubated at 60 °C for 1 h, deparaffinized in xylene, and rehydrated with graded alcohol washes. The slides were then washed three times with RNase-free PBS, digested with 8 mg/mL pepsin at 3 °C for 10 min, washed, and dehydrated in graded alcohol washes. The slides were hybridized overnight at 40 °C with 50 nM locked nucleic acid (LNA)-modified DIG-labelled probes for miR-106b-5p. After stringency washes (5×, 1×, and 0.2× saline-sodium citrate (SSC)), the slides were placed in blocking buffer for 30 min at room temperature, followed by overnight incubation at 4 °C in an alkaline phosphatase-conjugated anti-DIG Fab fragment solution. The antibody signal was developed with a 5-bromo-4-chloro-3-indolyl phosphate (*BCIP*)/nitro blue tetrazolium (*NBT*) substrate (Roche, Mannheim, Germany), and nuclei were stained with Nuclear Fast Red.

### Statistical analysis

Statistical analysis was performed with SPSS 19.0 software. All tests were two tailed, and *p* < 0.05 was considered statistically significant.

## Results

### CircMEMO1 expression was identified to be significantly Downregulated in HCC tissue and related to patient prognosis

**Fig. 1:**
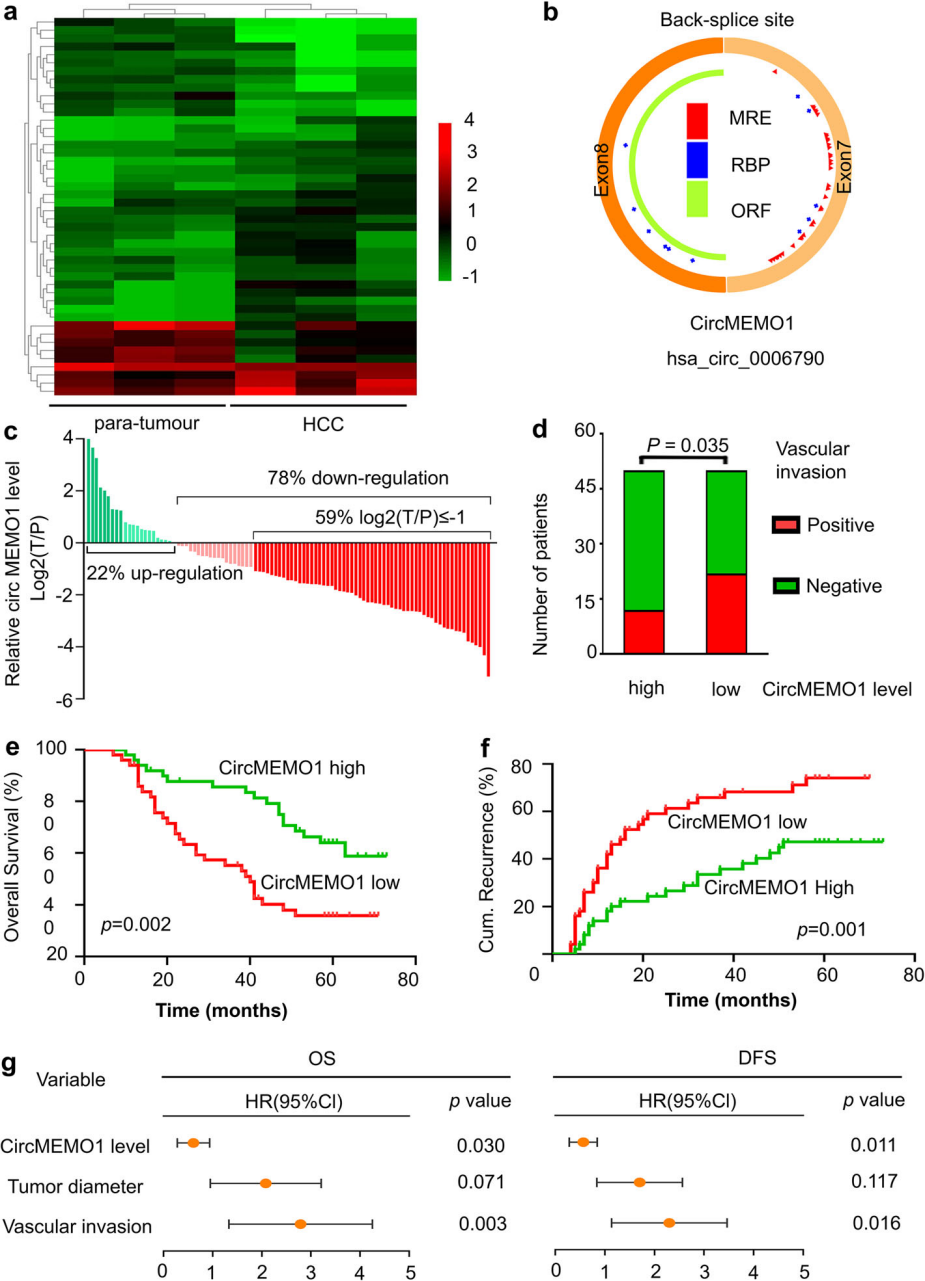
CircMEMO1 Expression Was Identified to Be Significantly Downregulated in HCC Tissue and Related to Patient Prognosis. **a** A heatmap of differentially expressed circRNAs in our HCC tissue samples compared with paratumour DN samples is shown. **b** Schematic illustration of circMEMO1. **c** qRT-PCR revealed that circMEMO1 expression was significantly downregulated in HCC tissue samples. **d** The circMEMO1 level in HCC tissue samples was related to vascular invasion in HCC patients. **e,f** Kaplan-Meier analysis showed that the level of circMEMO1 was predictive of overall survival and DFS in HCC. **g** Multivariate Cox analysis revealed that the circMEMO1 level in HCC tissue was an independent prognostic factor in HCC patients

First, the Arraystar Human circRNA Array combined with LCM was used to identify differentially expressed circRNAs in HCC tissue samples compared to paratumour DN samples. The circRNAs with significant differential expression (fold change ≥2.0 and *P* < 0.05) between the groups were identified. From the results of the Human circRNA Array analysis, 28 circRNAs were preliminarily identified to be upregulated, while 18 circRNAs were downregulated in the HCC tissue samples compared to the paratumour DN samples, and hsa_circ_0006790, called circMEMO1, was one of the most significantly differentially expressed circRNAs (Fig. 1a, S1a).

To clarify the characteristics of circMEMO1, we designed two pairs of circMEMO1-specific divergent primers to amplify the back-spliced products in HCC samples by qRT-PCR (circRNA sequencing data were obtained from the online database circBase). Our result suggested the expected length of the PCR products (Fig. 1b, S1b). Sanger sequencing of PCR products further confirmed the head-to-tail splicing in the PCR products with predicted splicing site (Fig. S1c). Besides, we treated the total RNA of Huh-7 cells with RNase R, and our results revealed that there was no change in circMEMO1 levels when the total RNA were treated with RNase R (Fig. S1d), indicating that the RNAs amplified by the specific primers were circular rather than linear.

To determine whether circMEMO1 was associated with HCC, we investigated the level of circMEMO1 in several HCC cell lines with different metastatic potential. The level of circMEMO1 was lower in the metastatic cell lines (MHCC97H, and HCCLM3) than in the non-metastatic lines (Hep3B, PLC/PRF/5, and Huh7) (Fig. S1e).

To confirm the change in circMEMO1 in HCC tissue, we detected the circMEMO1 level in a validation set (*n* = 100). Our results revealed that circMEMO1 was significantly downregulated in HCC tissue samples compared to paratumour samples (Fig. 1c, S1f). Then, we analysed the relationship between the circMEMO1 level and HCC patient prognosis. We found that the circMEMO1 level in HCC samples was related to vascular invasion in patients (Fig. 1d and Table S2). Kaplan-Meier survival curve analysis revealed that HCC patients with a high circMEMO1 level had significantly better OS and DFS than those with a low circMEMO1 level (Fig. 1e, f). Multivariate Cox analysis revealed that the circMEMO1 level in HCC tissue was an independent prognostic factor in HCC patients (Fig. 1g). These data suggest that the downregulated circMEMO1 level plays important roles in HCC tumorigenesis and progression.

### CircMEMO1 inhibits cell proliferation, migration and invasion in HCC cell lines in vitro and in vivo

**Fig. 2:**
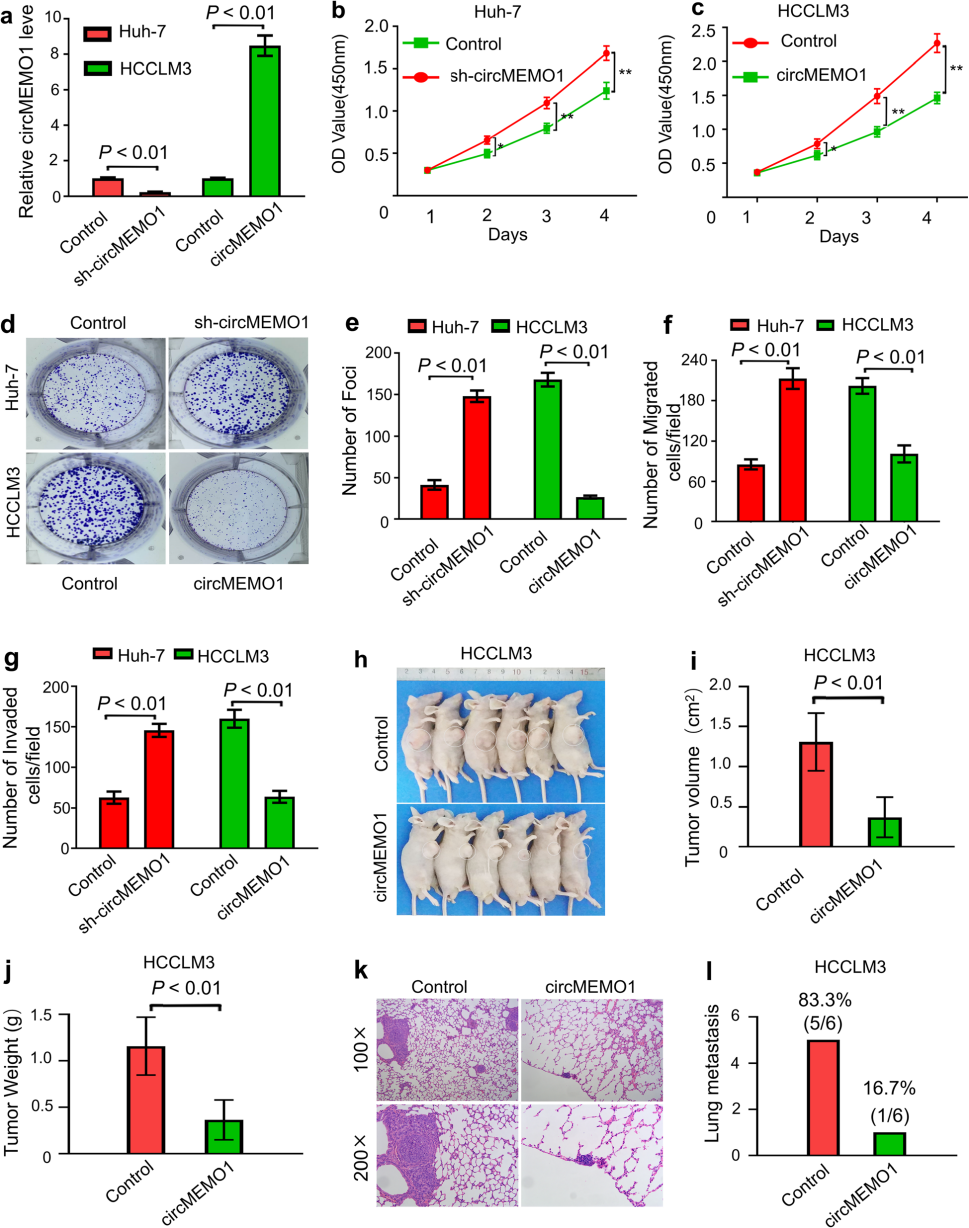
CircMEMO1 Inhibits HCC Cell Proliferation and Invasion In Vitro and In Vivo. **a** sh-circMEMO1 was overexpressed and knocked down in HCCLM3 and Huh-7 cells by lentivirus-mediated transduction, respectively. **b, c** Cell Counting Kit-8 (CCK-8) assays were used to determine the role of circMEMO1 in the proliferation of HCC cells. * *P* < 0.05; **, *P* < 0.01. **d, e** Colony formation assays showed that circMEMO1 inhibited the colony formation activity of HCC cells. **f, g** Transwell assays showed that circMEMO1 inhibited the migration and invasion of HCC cells. **h-j** Xenograft tumours composed of circMEMO1-OE HCCLM3 cells were significantly smaller than those composed of control cells. **k, l** circMEMO1 inhibited the lung metastasis of HCCLM3, and representative images of haematoxylin-eosin (HE) staining of metastatic nodules in the lungs of animals in different groups are shown

To investigate the biological function of circMEMO1 in HCC progression, circMEMO1 was stably overexpressed and knocked down in HCCLM3 and Huh-7 cells by lentivirus-mediated transduction, respectively (Fig. 2a). Cell proliferation, clonogenic and Transwell assays showed that the proliferative, clonogenic,and migration capacities of Huh-7 cells were significantly increased after circMEMO1 was knocked down, but that the proliferative, clonogenic, invasive and migration abilities of HCCLM3 cells were impaired when the circMEMO1 level was altered in vitro (Fig. 2b-g).

To further determine whether circMEMO1 serves as a tumour suppressor, we injected control HCCLM3 or circMEMO1-OE cells into nude mice. Our results revealed that 6 weeks after transplantation, the xenograft tumours composed of circMEMO1-OE HCCLM3 cells were significantly smaller than those composed of control cells (Fig. 2h-j). Only one of six HCCLM3-circMEMO1-OE xenograft mice (16.7%) had metastatic nodules in the lungs, whereas five of six control HCCLM3 xenograft mice (83.3%) had evidence of lung metastasis (Fig. 2k, l). Therefore, we speculated that circMEMO1 functions as a tumour suppressor to inhibit the proliferation, invasion and metastasis of HCC in vivo and in vitro.

### circMEMO1 regulates the level of the TET/5hmC Axis by sponging miR-106b-5p in HCC cells

**Fig. 3:**
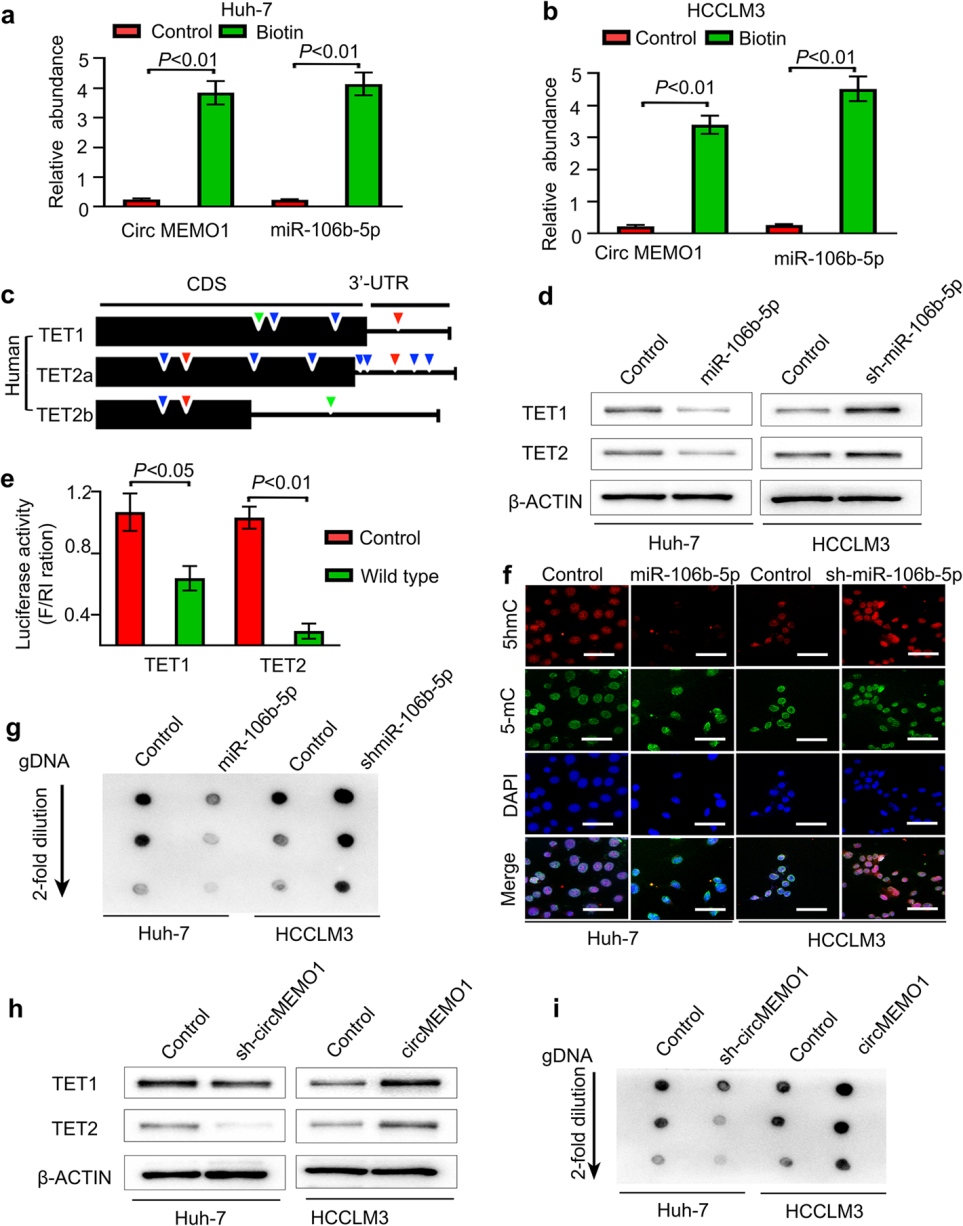
CircMEMO1 Regulates the Level of the TET1/5hmC Axis by Sponging MiR-106b-5p in HCC Cells. **a, b** miR-106b-5p was abundantly pulled down by a circMEMO1 probe, and miR-106b-5p captured large amounts of circMEMO1 in both Huh-7 and HCCLM3 cells. **c** Representative seed sequences for miR-106b-5p in the human TET family are shown: 9 base pairs (green), 8 base pairs (red) and 7 base pairs (blue). **d** HCC cancer cells with different miR-106b-5p expression levels were subjected to western blot analyses for the indicated proteins. **e** A luciferase assay with the luciferase gene linked to the 3′-UTR of TET1/2 was performed. HEK293 cells were transiently transfected with a combination of pGL3 firefly luciferase reporter plasmids encoding the wild-type 3′-UTR sequences of human TET genes, miR-106b-5p, and a Renilla luciferase reporter for normalization. The data are represented as the mean ± SD (*n* = 3). **f** 5hmC-, 5-mC- and DAPI-stained HCC cells expressing different miR-106b-5p levels are shown. Scale bars, 100 μm. **g** Genomic DNA purified from HCC cells with different miR-106b-5p levels was denatured and neutralized. Global 5hmC levels were then measured by using a dot blot assay with an anti-5hmC antibody. **h** HCC cancer cells with different circMEMO1 expression levels were subjected to western blot analyses for the indicated proteins. **i** A dot blot assay with an anti-5hmC antibody was used to detect changes in the 5hmC level in HCC cells with different circMEMO1 expression levels

CircRNAs have been reported to function as sponges for miRNAs [6, 15]. Then, we investigated the ability of circMEMO1 to function as a sponge for miRNAs. Candidate targets were determined using the target prediction engine starbase (starbase.sysu.edu.cn). Comparison of the predicted target information for circMEMO1 with miRNAs that were significantly upregulated in HCC tissue samples compared with paratumour tissue samples [16] revealed that miR-106b-5p potentially interacts with circMEMO1 (Fig. S2a). To investigate the direct interaction between circMEMO1 and miR-106b-5p, we designed a linear biotinylated circMEMO1 probe. Our qRT-PCR results showed that miR-106b-5p was abundantly pulled down by the circMEMO1 probe in both Huh-7 and HCCLM3 cells. In addition, we designed biotinylated miR-106b-5p to pull down circMEMO1 in Huh-7 and HCCLM3 cells. The qRT-PCR results revealed that miR-106b-5p captured circMEMO1 (Fig. 3a, b). However, circMEMO1 levels were not significantly changed in Huh-7 and HCCLM3 cells when miR-106b-5p was overexpressed or knocked down, while there were also no significant changes in miR-106b-5p after silencing or overexpressing circMEMO1 in HCC cells (Fig. S2b, c).

To further elucidate the molecular mechanism by which circMEMO1 inhibits HCC progression, we attempted to identify the target genes of miR-106b-5p. Previous studies revealed that among the three ten-eleven translocation (TET) genes, which encode the key dioxygenases that oxidize 5-position of cytosine (5-mC) into 5-hydroxymethylcytosine (5hmC), TET1 and TET2 have frequently been shown to exhibit downregulated expression in HCC tissue [17]. Based on online target prediction algorithms, we found that TET1 and TET2 were potential candidate target genes of miR-106b-5p (Fig. 3c, S2a).

To determine the changes in TET1 and TET2 expression in HCC cell lines expressing different levels of miR-106b-5p, we utilized pri-miR-106b-5p or small interfering RNA (siRNA) transduction via a lentivirus to upregulate or efficiently knock down miR-106b-5p expression in Huh-7 or HCCLM3 cells, respectively. Our results revealed that miR-106b-5p upregulation in Huh-7 cells dramatically suppressed TET1 and TET2 mRNA and protein levels, whereas inhibition of miR-106b-5p in HCCLM3 cells enhanced TET1 and TET2 mRNA and protein expression (Fig. 3d, S2d, e). Notably, a luciferase reporter assay was performed using the 3′-UTR of the TET1 or TET2 gene (Fig. 3e). In addition, mutant versions of miR-106b-5p miRNA recognition elements (MREs) indicated that the miR-106b-5p-mediated repression of TET proteins is due to direct interactions between miR-106b-5p and the genes (Fig. S2f).

Then, we detected changes in 5hmC and 5-mC levels to assess whether miR-106b-5p can remodel the epigenetic landscape by targeting TET genes and regulating 5hmC levels in the genome. As expected, Huh-7/miR-106b-5p-OE cells exhibited lower levels of 5hmC than Huh-7/Control cells, and inhibition of miR-106b-5p in HCCLM3 cells markedly enhanced the level of 5hmC, but the level of 5-mC was not changed (Fig. 3f, g). These results were in accordance with the results for 5hmC and 5-mC in HCC samples (Fig. S2g).

As circMEMO1 exerts biological function in HCC cells by sponging miR-106b-5p, we further detected changes in TET1 and TET2 protein expression and the gDNA 5hmC level in HCC cells with different circMEMO1 levels. Our results revealed that the down-regulation of circMEMO1 reduced TET1 and TET2 protein expression and the gDNA 5hmC level in Huh-7 cells, while overexpression of circMEMO1 increased TET1 and TET2 expression and the gDNA 5hmC level in HCCLM3 cells (Fig. 3h, i). These data indicate that circMEMO1 can sponge miR-106b-5p and regulate the TET/5hmc axis in HCC cells.

### The circMEMO1 level was correlated with the miR-106b-5p/TET1/5hmC axis and predicted a poor prognosis in HCC patients

**Fig. 4:**
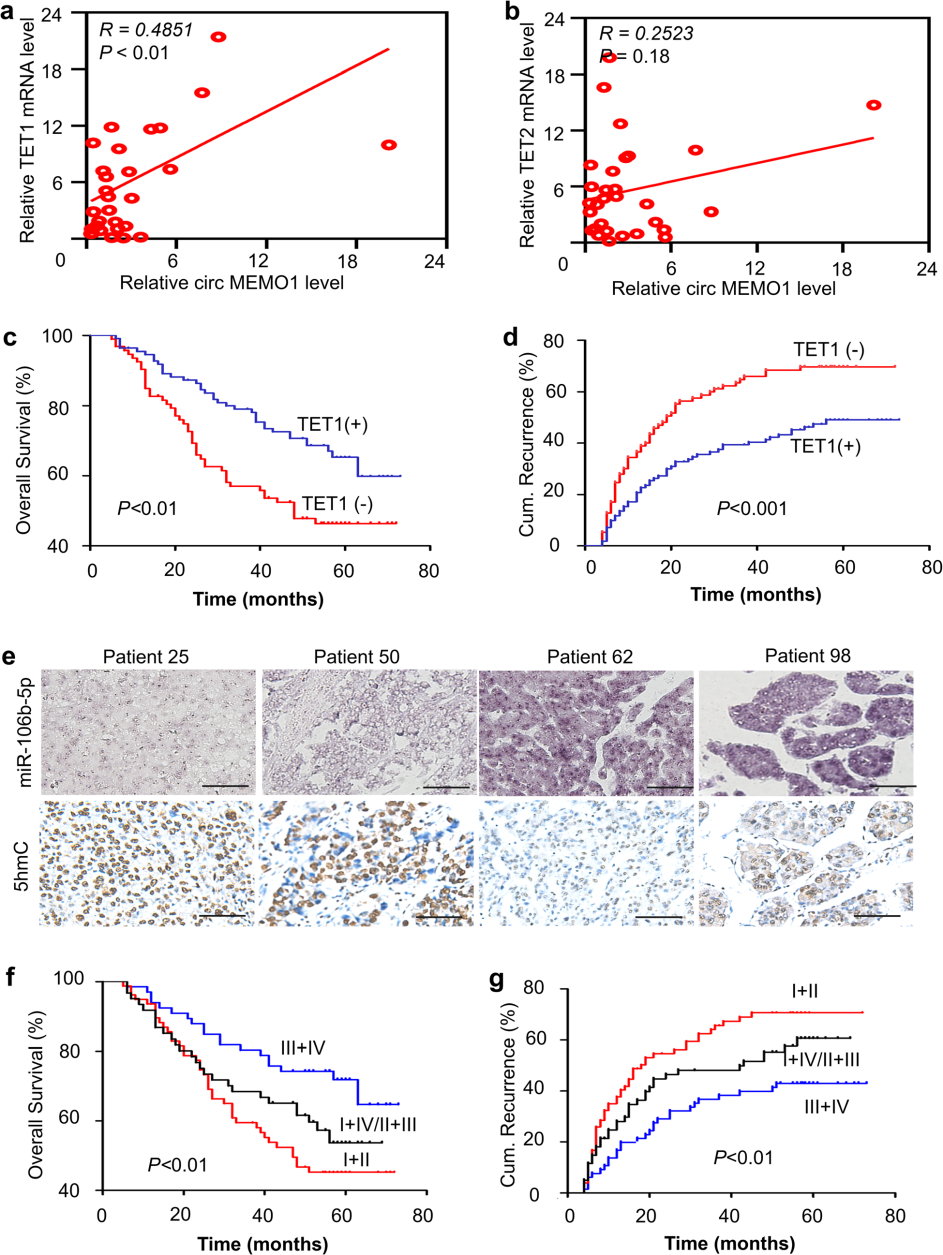
The circMEMO1 Level Correlates with the miR-106b-5p/TET1/5hmC Axis and Predicts a Poor Prognosis in HCC Patients. **a, b** The correlations between the level of circMEMO1 and mRNA levels of the TET1 and TET2 genes were analysed using *q*RT-PCR analysis of HCC patient samples. **c, d** HCC patient samples were subdivided into two groups according to the expression status of TET1. Kaplan-Meier plots representing the overall survival (c) and cumulative recurrence rate (d) of the stratified patients are shown. **e** Representative HCC cases in tissue microarrays (serial sections) were analysed by an in situ hybridization assay for miR-106b-5p and immunohistochemical staining for 5hmC. f, g Positive miR-106b-5p expression with a low level of 5hmC correlated with the worst survival (f) and highest recurrence rate (g) in HCC patients. I, miR-106b-5p (+); II, low 5hmC level; III, miR-106b-5p (−); IV, high 5hmC level

Then, we sought to evaluate whether our observations could be demonstrated in human HCC patients. First, we analysed the correlation between the levels of circMEMO1 and TET1/2 mRNA by qRT-PCR. We observed that the circMEMO1 level in human HCC samples was directly and positively correlated with the mRNA level of TET1 but not that of TET2 (Fig. 4a, b), which suggests that TET1 may play a critical role in HCC inhibition caused by circMEMO1.

To determine the role of TET1 protein in HCC progression and prognosis prediction in HCC patients, we further detected TET1 protein expression with IHC and a TMA. Critically, HCC patients found to have high levels of the TET1 protein had a better prognosis than those with low TET1 staining in terms of OS and a low cumulative recurrence rate (Fig. 4c, d). Multivariate Cox proportional hazards regression analyses revealed that TET1 was an independent prognostic factor in the HCC group (Tables S3, 4).

As miR-106b-5p and 5hmC serve as important intermediaries to mediate the biological function of circMEMO1 in regulating HCC progression, we further evaluated the relationship between the levels of miR-106b-5p and 5hmC in HCC patients using in situ hybridization and immunohistochemical staining, respectively. Our results revealed that HCC cells with high miR-106b-5p expression had a low level of 5hmC in human HCC samples, and vice versa (Fig. 4e). More importantly, HCC patients with high miR-106b-5p and low 5hmC staining had the worst prognosis in terms of OS and a high cumulative recurrence rate (Fig. 4f, g). Our data indicate that circMEMO1 may influence malignant cell properties in HCC and contribute to HCC progression by regulating the miR-106b-5p/TET1/5hmC axis.

### CircMEMO1 inhibits HCC metastasis and Stemness by regulating the EMT process via the MiR-106b-5p/TET1/5hmC Axis

Epithelial to mesenchymal transition (EMT) is a central process that is critical for the invasion and metastasis of cancer cells [18, 19]. In HCC, miR-106b-5p has been demonstrated to promote HCC migration and metastasis by activating the EMT process [20]. Our study found that circMEMO1 can sponge miR-106b-5p to inhibit HCC invasion and progression. These results prompted us to hypothesize that circMEMO1 may inhibit HCC metastasis by regulating the EMT process via the miR-106b-5p/TET1/5hmC axis.

**Fig. 5:**
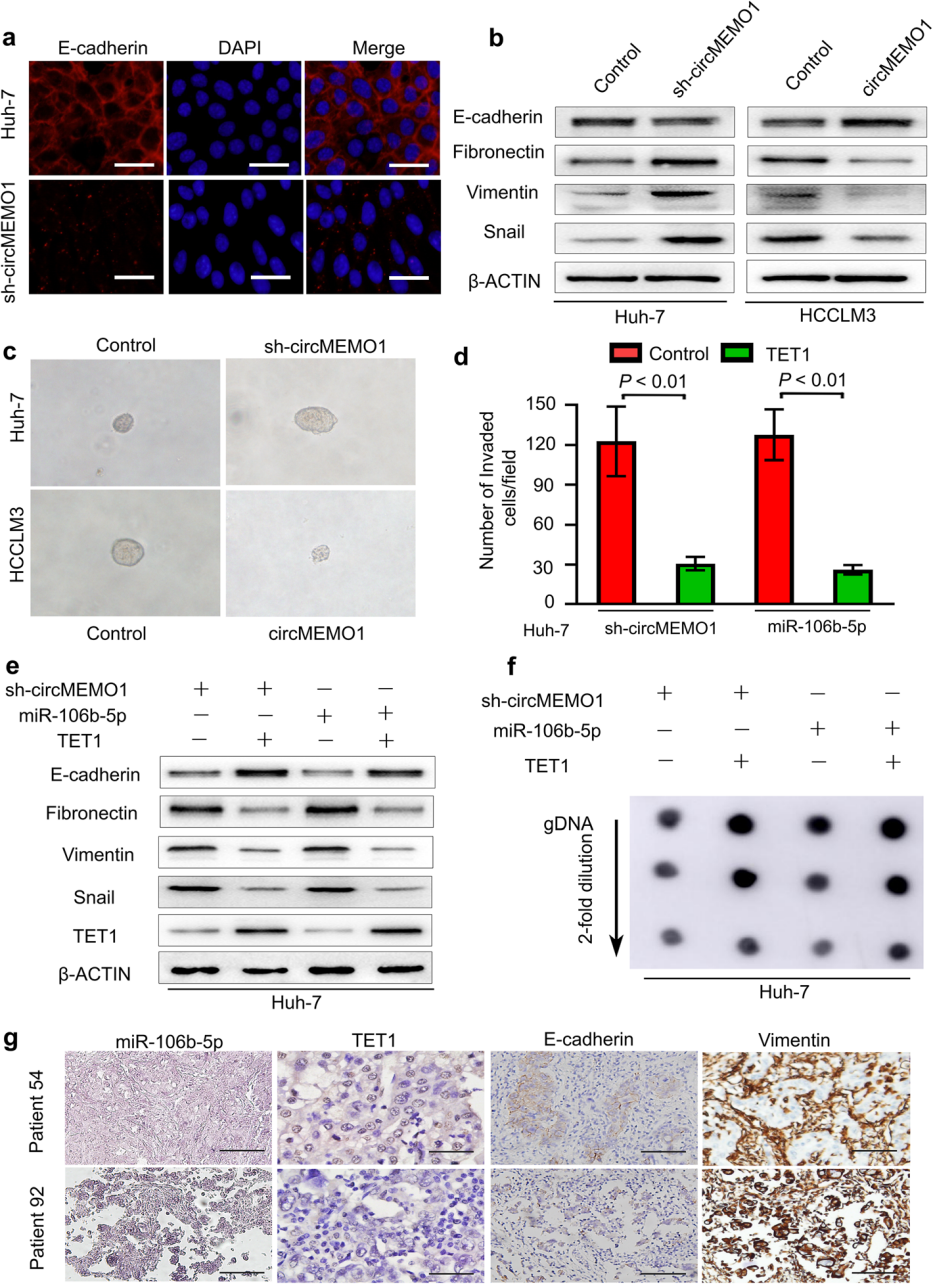
CircMEMO1 Inhibits HCC Progression by Regulating the EMT Process via the miR-106b-5p/TET1/5hmC Axis. **a** Huh-7 cells transduced with sh-circMEMO1 or an empty vector were subjected to immunofluorescence. Scale bars, 100 μm. **b** Western blotting was used to detect the levels of the indicated proteins in Huh-7 and HCCLM3 cells with different circMEMO1 levels. **c** sh-circMEMO1 expression increased the ability of Huh-7 cells to form tumoursphere structures, while overexpression of circMEMO1 expression decreased the ability of HCCLM3 cells to form tumoursphere structures. **d** Huh-7 cells infected with a combination of sh-circMEMO1, miR-106b-5p, and TET1-expressing vectors were subjected to a cell invasion assay. Scale bars, 100 μm. **e** Cell lysates from Huh-7 cells expressing a combination of sh-circMEMO1, miR-106b-5p, and TET1 were subjected to western blot analysis for the indicated proteins. **f** Genomic DNA purified from Huh-7 cells expressing a combination of sh-circMEMO1, miR-106b-5p, and TET1 was denatured and neutralized. Global 5hmC levels were then measured by a dot blot assay using an anti-5hmC antibody. **g** Representative HCC cases in tissue microarrays were analysed by an in situ hybridization assay for miR-106b-5p and immunohistochemical staining for TET1, E-cadherin and Vimentin

To confirm our hypothesis, we investigated the expression of molecular markers related to EMT in HCC cells. Compared with counterpart cells, sh-circMEMO1 HCC cells exhibited weaker expression of the epithelial marker E-cadherin and stronger expression of the mesenchymal markers Fibronectin, Vimentin and Snail. Additionally, western blotting results confirmed that upregulation of circMEMO1 expression in HCCLM3 cells decreased the expression of the mesenchymal markers Fibronectin and Vimentin and the transcription factor Snail but increased that of the epithelial marker E-cadherin (Fig. 5a, b).

The EMT process is known to be correlated with normal and malignant mammary stem cell function [21, 22]. We further speculated that circMEMO1 may also inhibit HCC cell stemness. Notably, the down-regulation of circMEMO1 expression could increase the ability of Huh-7 cells to form tumoursphere structures, while overexpression of circMEMO1 decreased the ability of HCCLM3 cells to form tumoursphere structures (Fig. 5c).

As circMEMO1 was directly and positively correlated with the TET1 mRNA level in human HCC samples, we further attempted to validate whether the TET1/5hmC axis plays a direct role in the function of circMEMO1 or miR-106b-5p. First, TET1 was ectopically expressed in Huh-7/sh-circMEMO1 or Huh-7 miR-106b-5p-OE cells, and then the invasive ability of these cells was analysed. We found that exogenously expressed TET1 protein significantly reduced the enhanced cancer cell invasion mediated by sh-circMEMO1 or miR-106b-5p overexpression (Fig. 5d). In addition, ectopic expression of TET1 also increased E-cadherin expression and the global 5hmC level but decreased the expression of fibronectin, vimentin and Snail (Fig. 5e, f). These results suggest that the TET1/5hmC axis plays an important role in the functions of miR-106b-5p and circMEMO1 in HCC cells.

To further elucidate the clinical significance of the miR-106b-5p/TET1/E-cadherin axis in HCC patients, we also detected the expression of TET1, E-cadherin and vimentin via immunohistochemical staining of a TMA of HCC specimens. We found that positive miR-106b-5p expression was significantly correlated with low levels of TET1 in tumour specimens, with the tumour specimens positive for miR-106b-5p usually presenting low E-cadherin and high Vimentin expression (Fig. 5g**)**. These data confirmed that circMEMO1 inhibits HCC metastasis and stemness by regulating the EMT process via the miR-106b-5p/TET1/5hmC axis.

### CircMEMO1 inhibits HCC progression by regulating the DNA Demethylation and expression of TCF21

TET enzymes catalyse the oxidation of 5-mC to 5hmC, leading to DNA demethylation and gene expression regulation. As TET1 can be guided by TARID-mediated R-loop formation to the TCF21 promoter to increase demethylation and activate transcription [23, 24], we hypothesize that circMEMO1 and miR-106b-5p may modulates the demethylation of the promoter within the TCF21 gene and regulate its expression, leading to the changes of its cognate targets.

**Fig. 6:**
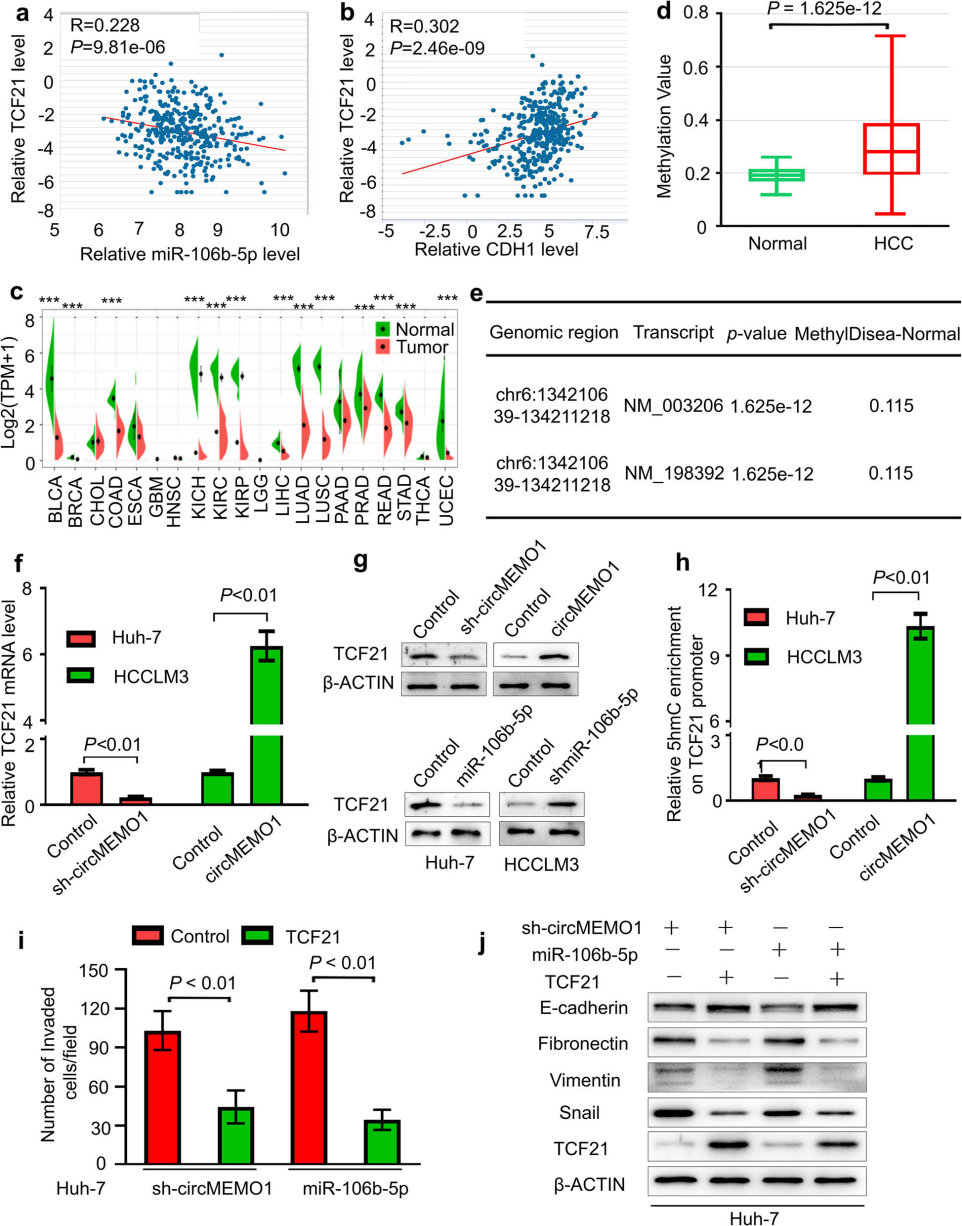
CircMEMO1 Inhibits HCC progression by Regulating the DNA demethylation and expression of TCF21. **a, b** The relationship of the TCF21 mRNA level with miR-106b-5p or CDH1 level in TCGA database. **c** The TCF21 mRNA level was downregulated in a series of cancers, including HCC, compared with normal tissues. **d, e** The TCF21 gene promoter methylation level between the HCC tissues and the controls was significantly higher in HCC patients than in controls from the same research. **f, g** circMEMO1 and miR-106b-5p can regulate TCF21 expression. **h** hMeDIP-qPCR assays showed that TCF21 genes had decreased 5hmC in gene bodies in Huh-7 and HCCLM3 cells with different circMEMO1 levels. **i, j** TCF21 overexpression diminished the metastasis properties and EMT process induced by inhibition of circMEMO1 or activation of miR-106b-5p

To address this possibility, we first evaluated the relationship of TCF21 and miR-106b-5p/CDH1 levels in HCC samples in TCGA database. Our results revealed that the TCF21 mRNA level was negatively correlated with the miR-106b-5p level but positively correlated with the CDH1 level (Fig. 6a, b). In addition, the TCF21 mRNA level was downregulated in a series of cancers, including HCC, compared with normal tissues (Fig. 6c, S3a, b), and its level in HCC samples was related to HCC patient prognosis (Fig. S3c, d). More importantly, the TCF21 gene promoter methylation level between the HCC samples was significantly higher in HCC patients than in controls from the same research, according to DiseaseMeth version 2.0 (Fig. 6d, e). Therefore, TCF21 may function as an important tumour suppressor and was downregulated in HCC samples due to aberrant DNA promoter methylation.

We further detected the expression of TCF21 in HCC cells with different circMEMO1 or miR-106b-5p levels. We found that circMEMO1 can promote the mRNA and protein expression of TCF21, while miR-106b-5p can inhibit the TCF21 level in HCC cells (Fig. 6f, g). The results of hMeDIP-qPCR assays showed that TCF21 genes show diferent 5hmC in gene bodies in Huh-7 and HCCLM3 cells with different circMEMO1 levels (Fig. 6h). More importantly, TCF21 overexpression diminished the metastasis promotion and EMT process induced by inhibition of circMEMO1 or activation of miR-106b-5p (Fig. 6i, j).

These results demonstrated that circMEMO1 inhibits HCC progression by regulating the DNA promoter demethylation and expression of TCF21.

### The circMEMO1 level in HCC cells regulated the antitumour activity of Sorafenib treatment

Currently, sorafenib remains an important approved standard treatment for advanced HCC patients. The rapid development of acquired resistance results in sorafenib treatment with limited clinical efficacy [3]. Recent studies have reported that cancer cells possessing EMT properties during the progression of HCC are capable of sorafenib resistance [25, 26]. Our study revealed that the circMEMO1/miR-106b-5p/TCF21 axis modulates HCC progression by regulating the EMT process. It is possible that the level of circMEMO1 in HCC cells could influence sensitivity to sorafenib treatment.

**Fig. 7:**
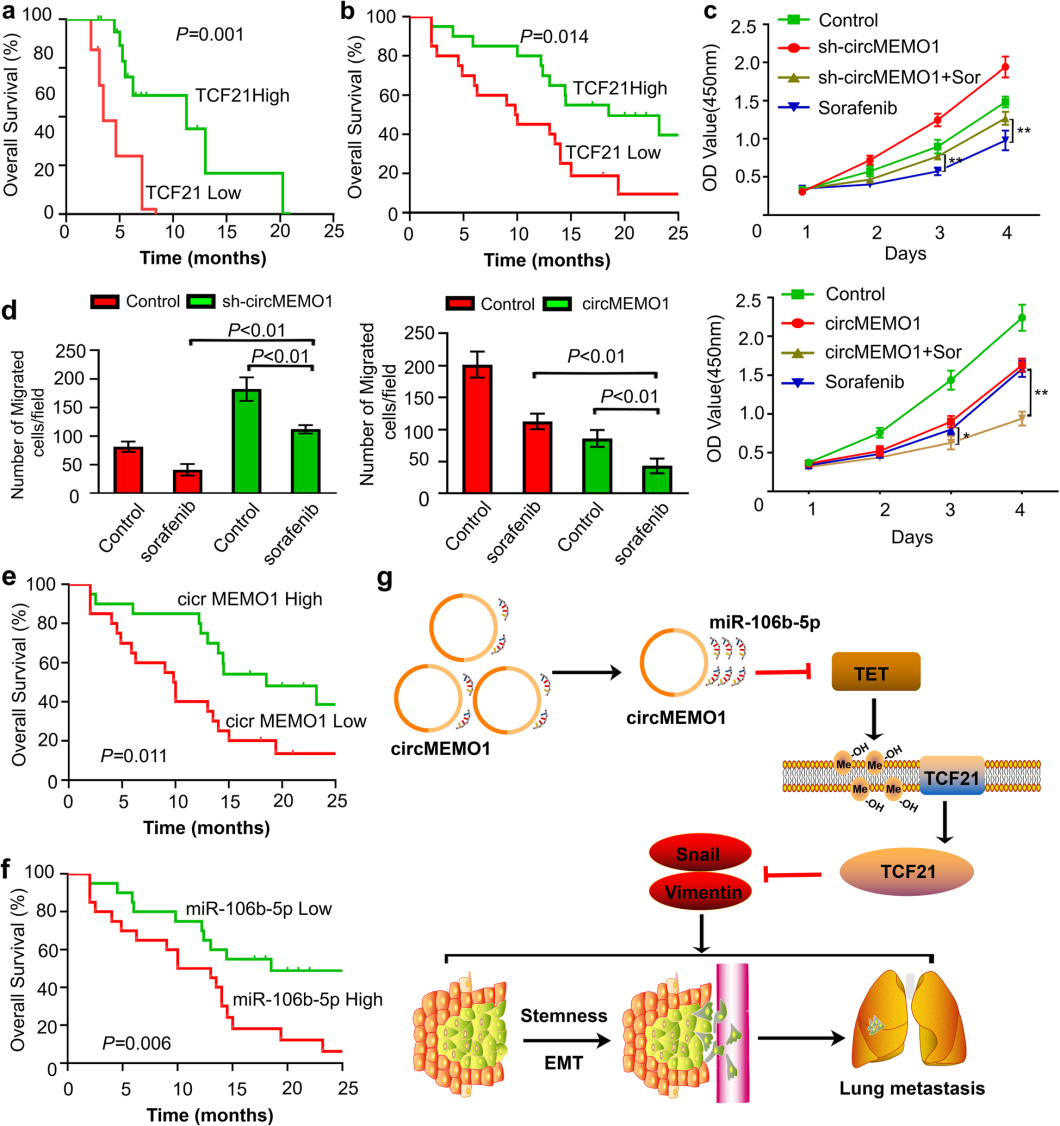
CircMEMO1 Regulated the Antitumour Activity of Sorafenib Treatment in HCC Cells. **a** Overall survival curves were compared between HCC patients with a high or low TCF21 level treated with sorafenib in TCGA database. **b** Comparison of overall survival curves between 40 advanced-stage recurrent HCC patients receiving combined sorafenib and TACE treatment who underwent liver resection before combined therapy. **c** MTT assays were used to detect the growth of Huh-7 and HCCLM3 cells with different circMEMO1 levels treated with sorafenib. **d** Matrigel invasion assays were used to investigate the migration ability of HCC cells with different circMEMO1 levels treated with sorafenib. **e, f** Overall survival curves were compared between recurrent HCC patients with a high or low circMEMO1 or miR-106b-5p level treated with sorafenib. **g** Schematic depiction of the mechanisms underlying circMEMO1 regulating HCC progression via the miR-106b-5p/TET1/5hmC axis

To validate our hypothesis, we first evaluated the TCF21 level and the prognosis of HCC patients treated with sorafenib. The analytic results in the TGCA database revealed that the TCF21 level was correlated with OS in HCC patients treated with sorafenib (Fig. 7a). Then, we analysed retrospective data from 40 advanced-stage recurrent HCC patients receiving combined sorafenib and TACE treatment who underwent liver resection before combined therapy. Our results also suggested that TCF21 level was also correlated with those patients prognosis (Fig. 7b).

Then, we detected the influence of the circMEMO1 level on the growth and migration ability changes of HCC cells treated with sorafenib in vitro. MTT results showed that downregulation of circMEMO1 expression could weaken the growth inhibition induced by 10 mmol/L sorafenib treatment in HCC cells, while overexpression of circMEMO1 could enhance the growth inhibition induced by sorafenib treatment (Fig. 7c). Matrigel assays indicated that Huh-7 cells with lower circMEMO1 levels had an enhanced migration ability compared with control Huh-7 cells, whereas the opposite result was seen when circMEMO1 was overexpressed in HCCLM3 cells (Fig. 7d).

To further determine the role of circMEMO1 in regulating the sensitivity of sorafenib treatment, the levels of circMEMO1 and miR-106b-5p were detected in HCC tissue samples from the above 40 advanced-stage recurrent HCC patients. Kaplan-Meier survival analysis showed that the OS of the circMEMO1-low group was much worse than that of the circMEMO1-high group, while the OS of the miR-106b-5p-low group was much better than that of the miR-106b-5p-high group (Fig. 7e, f). Thus, we conclude that circMEMO1 and its downstream target miR-106b-5p can regulate the sensitivity of HCC cells to sorafenib treatment.

## Discussion

Here, we report that circMEMO1 can modulate the promoter methylation and expression of TCF21 to regulate HCC progression and sensitivity to sorafenib treatment via the miR-106b-5p/TET1/5hmC axis. First, the Arraystar Human circRNA Array combined with laser capture microdissection (LCM) was used to identify that circMEMO1 was significantly downregulated in HCC tissue samples and that the level of circMEMO1 was closely related to the OS and disease-free survival (DFS) of HCC patients. Ectopic expression of circMEMO1 led to inhibition of cell motility, metastasis and stemness in HCC cells via the EMT process. Mechanistic analysis revealed that circMEMO1 can promote the demethylation process of the TCF21 promoter, which is very frequently hypermethylated in HCC tissues, and then promote the transcriptional activation and expression of TCF21 by acting as a sponge for miR-106b-5p, which targets TET family genes and increases the 5hmC level. More importantly, circMEMO1 can increase the sensitivity of HCC cells to sorafenib treatment. Therefore, circMEMO1 can promote the demethylation process and increase the expression of TCF21 and can be considered a crucial epigenetic modifier and an important tumour suppressor in HCC progression.

The EMT process executed by cancer cells is one of multiple events needed for invasion and migration. Cancer cells undergoing EMT tend to detach from the primary tumour, invade through the extracellular matrix, and migrate to form distant metastases upon recognition of certain cues in the tumour microenvironment [19–22]. In addition, the EMT process is thought to be a developmental programme instrumental to the acquisition of stemness by tumour cells that then leads to the generation of CSCs [21, 22]. Elucidating the molecular mechanism that regulates the EMT process in HCC cells is important for inhibiting HCC metastasis and improving patient prognosis. Our results demonstrated that circMEMO1 inhibited HCC invasion, metastasis and stemness by regulating the miR-106b-5p/TET1/5hmC axis and EMT process. Overexpression of circMEMO1 restored the expression of E-cadherin, decreased the expression of mesenchymal components in vitro, and inhibited lung metastasis of HCC cells in vivo. Ectopic TET1 protein expression significantly reduced the enhanced cancer cell invasion and mesenchymal component expression and increased the weakened E-cadherin expression and global 5hmC level induced by sh-circMEMO1 or miR-106b-5p. Therefore, circMEMO1 inhibits HCC metastasis and stemness by regulating the miR-106b-5p/TET1/5hmC axis and antagonizing the EMT process.

As an important regulator in tumorigenesis and cancer metastasis, aberrant DNA methylation in the regulatory region of cancer-associated genes, which can be dynamically altered by new epigenetic programmes, has been established as an alternative mechanism to heritably silence gene transcription. As an intermediate base, 5hmC plays unique roles in the control of DNA promotion of the methylation process, gene expression and pathogenesis of cancer [27, 28], and its level in cancer cells is associated with downregulation of the expression of the TET (ten-eleven translocation) family of proteins that are strongly enriched at gene promoters, mainly catalysing the hydroxylation of DNA 5-mC into 5hmC in CpG islands, maintaining the fidelity of DNA methylation patterns by mediating demethylation, and then likely altering the local chromatin environment to activate gene transcription [29, 30]. Previous studies have reported that TET family genes are silenced through genetic mutation and blockade of catalytic activity via removal of the obligate oxygen substrate under hypoxic conditions [31, 32] and are considered to be genuine tumour suppressor genes. However, the mechanism of TET/5hmC inactivation and whether the TET/5hmC axis is involved in the regulation of HCC stemness and treatment sensitivity remain unresolved. Here, we identified circMEMO1 as an important noncoding RNA that regulates the expression of the TET family of genes and the 5hmC level to restrain HCC progression. Mechanistically, circMEMO1 serves as the sponge of oncogenic miR-106b-5p, which binds to the 3′-UTR of TET mRNAs to strongly decrease their expression in HCC cells and downregulate 5hmC levels. In HCC samples, there was a positive correlation between circMEMO1 and TET1 expression. Ectopic TET1 expression rescued the reduced level of 5hmC induced by sh-circMEMO1 or miR-106b-5p in HCC cells. These data support the conclusion that circMEMO1 and miR-106b-5p can regulate the level of the TET1/5hmC axis and act as key epigenetic modifiers in HCC.

## Conclusion

We identified circMEMO1 as a crucial tumour suppressor in HCC metastasis and stemness that functions via the miR-106b-5p/TET1/5hmC/TCF21 axis and EMT process. As a key epigenetic modifier, circMEMO1 can also regulate the sensitivity of HCC to sorafenib treatment.

## Supplementary Information

**Additional file 1: Figure S1** CircMEMO1 Expression Was Identified to Be Significantly Downregulated in HCC Tissue and Related to Patient Prognosis. **a** The number of differentially expressed circRNAs in our HCC tissue samples compared with paratumour DN samples is shown. **b** PCR validation of the circ MEMO1 amplified by divergent primers using the template cDNA derived from HCC samples. **c** The Sanger sequencing of the back-splice sites of the products from B. **d** qRT-PCR analysis of circMEMO1 expression in HCC cell lines was performed. **e** qRT-PCR results showed the changes after RNase R treatment. **f** qRT-PCR revealed that circMEMO1 expression was significantly downregulated in HCC tissue samples.

**Additional file 2: Figure S2** CircMEMO1 Regulates the Level of the TET1/5hmC Axis by Sponging MiR-106b-5p in HCC Cells. **a** miR-106b-5p potentially interacted with circMEMO1, and TET1 and TET2 were potential candidate target genes of miR-106b-5p. **b** There were no significant changes in circMEMO1 following overexpression or knockdown of miR-106b-5p in Huh-7 and HCCLM3 cells. **c** There were no significant changes in miR-106b-5p after silencing or overexpressing circMEMO1 in HCC cells. **d, e** miR-106b-5p regulated the level of TET1 and TET2 mRNA in HCC cells. **f** Luciferase assay of mutant versions of miR-106b-5p MREs linked to the 3′-UTR of TET1/2. **g** 5hmC and 5mC levels were analysed by immunofluorescence with sections obtained from HCC patient sample.

**Additional file 3: Figure S3** The TCF21 level was downregulated in HCC samples and was related to HCC patient prognosis. **a, b** TCGA database alone or combined with GTEx database showed that the TCF21 mRNA level was downregulated in HCC samples. **c, d** TCGA database analysis showed that the TCF21 level in HCC samples was related to HCC patient prognosis.

**Additional file 4: Table S1** The Sequences of RT-PCR Primers

**Additional file 5: Table S2** Association of Circular RNA MEMO1 Expression with Clinicopathological Parameters of HCC Patients

**Additional file 6: Table S3** Univariate and Multivariate Analysis of Prognostic Factors of DFS

**Additional file 7: Table S4** Univariate and Multivariate Analysis of Prognostic Factors of OS

**Additional file 8.** Materials and Methods.

## Data Availability

All data generated or analyzed during this study are included either in this article or in the supplementary Materials and Methods, Tables, Figures, and Figure Legends files.

## Abbreviations

HCC: Hepatocellular carcinoma
DN: Dysplastic nodules
circRNAs: Circular RNAs
TCF21: Transcription factor 21
LCM: Laser Capture Microdissection
TET: Ten-eleven translocation
*q*RT-PCR: Quantitative real-time polymerase chain reaction
UTR: Untranslated region
TMA: Tissue microarray
FFPE: Formalin-fixed paraffin-embedded
MRE: Micro-RNA recognition element
5-mC: 5-position of cytosine
5hmC: 5-hydroxymethylcytosine
IHC: Immunohistochemistry
EMT: Epithelial to mesenchymal transition
shRNA: Short hairpin RNA
DMEM: Dulbecco’s modified Eagle medium
FBS: Fetal bovine serum
PBS: Phosphate buffer solution
BSA: Bovine serum albumin
DAPI: 4′,6-Diamidino-2-phenylindole
SDS: Sodium dodecyl sulfate
OS: Overall survival
DFS: Disease-free survival
