# Supplementary figures and images for: CircMEMO1 modulates the promoter methylation and expression of TCF21 to regulate hepatocellular carcinoma progression and sorafenib treatment sensitivity
Source: Mol Cancer. 2021 May 13;20:75. doi: 10.1186/s12943-021-01361-3 (PMC8117652; doi:10.1186/s12943-021-01361-3)

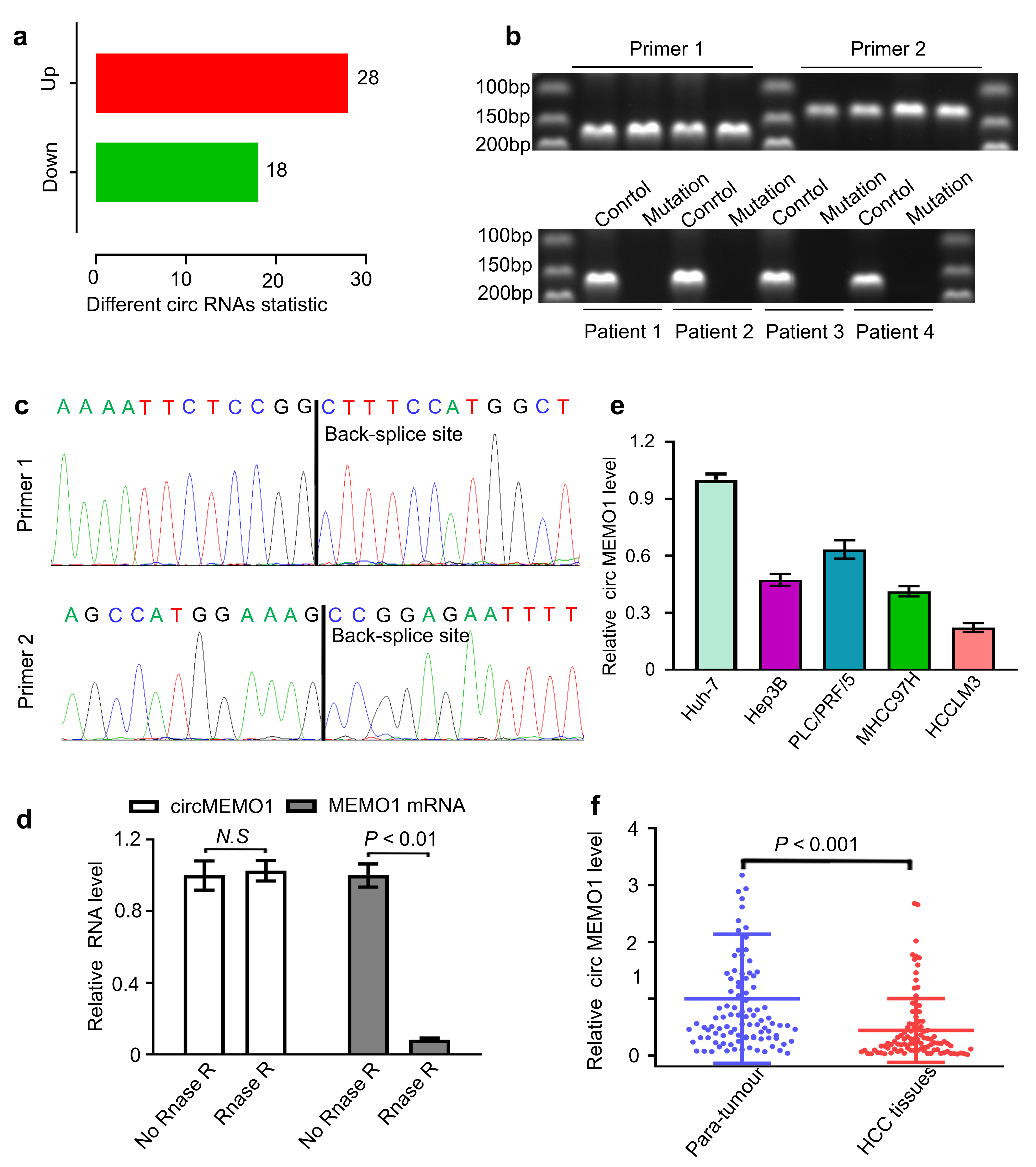

Supplement: Supplementary file 1 — Additional file 1: Figure S1 CircMEMO1 Expression Was Identified to Be Significantly Downregulated in HCC Tissue and Related to Patient Prognosis. a The number of differentially expressed circRNAs in our HCC tissue samples compared with paratumour DN samples is shown. b PCR validation of the circ MEMO1 amplified by divergent primers using the template cDNA derived from HCC samples. c The Sanger sequencing of the back-splice sites of the products from B. d qRT-PCR analysis of circMEMO1 expression in HCC cell lines was performed. e qRT-PCR results showed the changes after RNase R treatment. f qRT-PCR revealed that circMEMO1 expression was significantly downregulated in HCC tissue samples. [file 12943_2021_1361_MOESM1_ESM.tif]

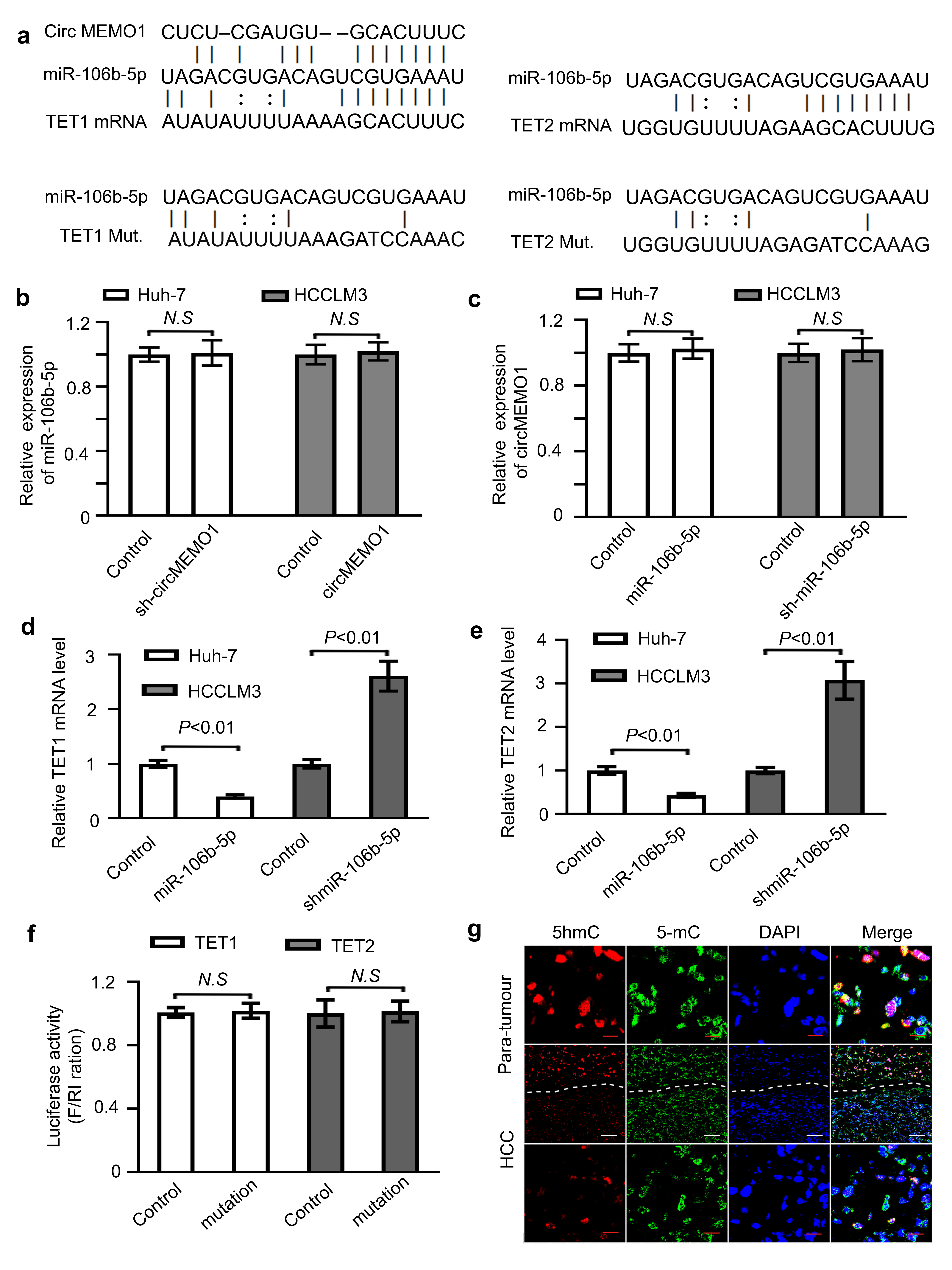

Supplement: Supplementary file 2 — Additional file 2: Figure S2 CircMEMO1 Regulates the Level of the TET1/5hmC Axis by Sponging MiR-106b-5p in HCC Cells. a miR-106b-5p potentially interacted with circMEMO1, and TET1 and TET2 were potential candidate target genes of miR-106b-5p. b There were no significant changes in circMEMO1 following overexpression or knockdown of miR-106b-5p in Huh-7 and HCCLM3 cells. c There were no significant changes in miR-106b-5p after silencing or overexpressing circMEMO1 in HCC cells. d, e miR-106b-5p regulated the level of TET1 and TET2 mRNA in HCC cells. f Luciferase assay of mutant versions of miR-106b-5p MREs linked to the 3′-UTR of TET1/2. g 5hmC and 5mC levels were analysed by immunofluorescence with sections obtained from HCC patient sample. [file 12943_2021_1361_MOESM2_ESM.tif]

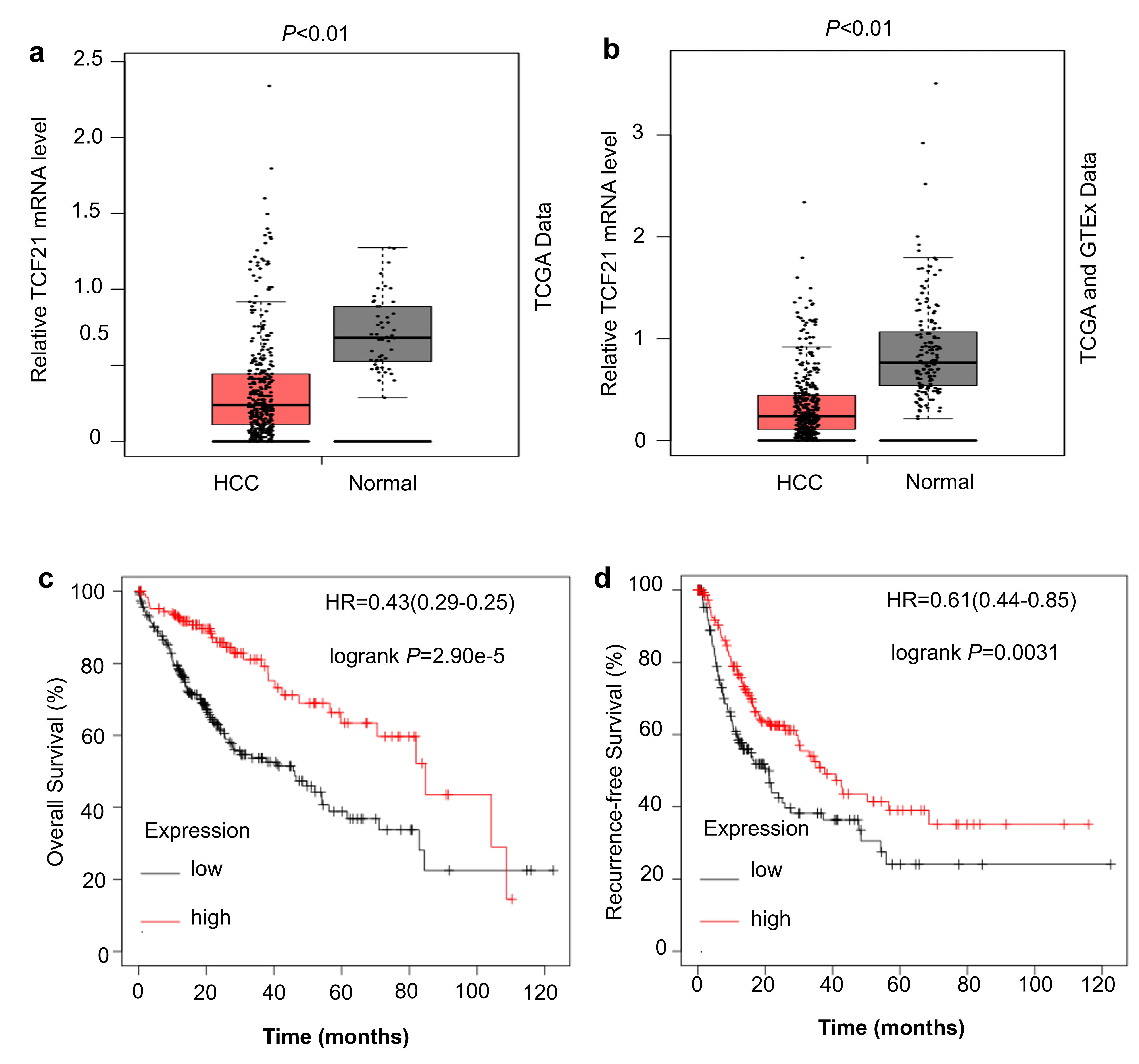

Supplement: Supplementary file 3 — Additional file 3: Figure S3 The TCF21 level was downregulated in HCC samples and was related to HCC patient prognosis. a, b TCGA database alone or combined with GTEx database showed that the TCF21 mRNA level was downregulated in HCC samples. c, d TCGA database analysis showed that the TCF21 level in HCC samples was related to HCC patient prognosis. [file 12943_2021_1361_MOESM3_ESM.tif]
