# Supplementary material for: CircMEMO1 modulates the promoter methylation and expression of TCF21 to regulate hepatocellular carcinoma progression and sorafenib treatment sensitivity
Source: Mol Cancer. 2021 May 13;20:75. doi: 10.1186/s12943-021-01361-3 (PMC8117652; doi:10.1186/s12943-021-01361-3)
Supplement: Supplementary file 4 — Additional file 4: Table S1 The Sequences of RT-PCR Primers [file 12943_2021_1361_MOESM4_ESM.docx]

### TABLE S1. The Sequences of RT-PCR Primers

| **Genes** |  | **Sequences** | **Length (bp)** |
| --- | --- | --- | --- |
| Circ MEMO1 | F1 | TAAAGCCATGGAAAGCCGGAGAA | 166 |
|  | R1 | GCGTTCAAACATTCCTGTCTTCC |  |
|  | F1 Mut. | ATACACTGAAAGGGACCGGAGAA |  |
|  | F2 | GATGAAAATTCTCCGGCTTTC | 142 |
|  | R2 | CGGAGAACTGTGGAAGACAG |  |
| MEMO1 | F | TTGGGCCTTCTCATCATGTGC | 135 |
|  | R | TGCGTTCAAACATTCCTGTCT |  |
| TET1 | F | GCTATACACAGAGCTCACAG | 139 |
|  | R | GCCAAAAGAGAATGAAGCTCC |  |
| TET2 | F | CTTTCCTCCCTGGAGAACAGCTC | 146 |
|  | R | TGCTGGGACTGCTGCATGACT |  |
| TCF21 | F | CATTCACCCGGTCAACCT | 62 |
|  | R | TCAGGTCACTCTCGGGTTTC |  |
| Actin | F | CATGTACGTTGCTATCCAGGC | 280 |
|  | R | CTCCTTAATGTCACGCACGAT |  |
