# Supplementary material for: CircMEMO1 modulates the promoter methylation and expression of TCF21 to regulate hepatocellular carcinoma progression and sorafenib treatment sensitivity
Source: Mol Cancer. 2021 May 13;20:75. doi: 10.1186/s12943-021-01361-3 (PMC8117652; doi:10.1186/s12943-021-01361-3)
Supplement: Supplementary file 5 — Additional file 5: Table S2 Association of Circular RNA MEMO1 Expression with Clinicopathological Parameters of HCC Patients [file 12943_2021_1361_MOESM5_ESM.docx]

**TABLE S2 Association of Circular RNA MEMO1 Expression with Clinicopathological Parameters of HCC Patients**

| **Clinicopathological Parameters** | **Total** | **High Circular RNA MEMO1 level** | | |
| --- | --- | --- | --- | --- |
|  |  | **N (%)** | **χ2** | ***p* value** |
| **Sex** | 100 |  |  |  |
| **Male** | 82 | 40(48.78) | 0.271 | 0.603 |
| **Femal** | 18 | 10 (55.56) |  |  |
| **Age range,yr** |  |  | 0.040 | 0.841 |
| **≤ 53** | 49 | 25 (51.02) |  |  |
| **﹥53** | 51 | 25(49.02) |  |  |
| **Tumor size, cm** |  |  | 5.760 | 0.016 |
| **≤5** | 50 | 31(62.00) |  |  |
| **>5** | 50 | 19(38.00) |  |  |
| **Tumor number** |  |  | 1.084 | 0.298 |
| **1** | 82 | 43(52.44) |  |  |
| **≥2** | 18 | 7(38.89) |  |  |
| **Grade of differentiation** |  |  | 0.053 | 0.817 |
| **I-II** | 75 | 37(49.33) |  |  |
| **III-IV** | 25 | 13(52.00) |  |  |
| **Vascular invasion** |  |  | 4.456 | 0.035 |
| **Yes** | 34 | 12 (35.29) |  |  |
| **No** | 66 | 38 (57.58) |  |  |
| **Tumor capsule** |  |  | 0.644 | 0.422 |
| **None** | 54 | 25 (46.30) |  |  |
| **Yes** | 46 | 25(54.35) |  |  |
| **Cirrhosis** |  |  | 0.162 | 0.687 |
| **Mild** | 56 | 27(48.21) |  |  |
| **Severe** | 44 | 23(52.27) |  |  |
| **AFP, ng/ml** |  |  | 4.456 | 0.035 |
| **≤400** | 66 | 38(57.58) |  |  |
| **>400** | 34 | 12(35.29) |  |  |
