# Supplementary material for: CircMEMO1 modulates the promoter methylation and expression of TCF21 to regulate hepatocellular carcinoma progression and sorafenib treatment sensitivity
Source: Mol Cancer. 2021 May 13;20:75. doi: 10.1186/s12943-021-01361-3 (PMC8117652; doi:10.1186/s12943-021-01361-3)
Supplement: Supplementary file 6 — Additional file 6: Table S3 Univariate and Multivariate Analysis of Prognostic Factors of DFS [file 12943_2021_1361_MOESM6_ESM.docx]

**TABLE S3 Univariate and Multivariate Analysis of Prognostic Factors of DFS**

| **Variable** | **Univariate** | | **Multivariate** | |
| --- | --- | --- | --- | --- |
|  | **χ2** | **p value** | **HR(95%Cl)** | **p value** |
| **TET1 expression (postive vs negative)** | 11.934 | 0.001 | 0.424-0.905 | 0.013 |
| **Tumor diameter (>5 cm vs ≤5 cm)** | 9.464 | 0.002 | 1.136-2.404 | 0.009 |
| **Tumor number (≥2 vs 1)** | 6.302 | 0.012 | 0.900-2.355 | 0.125 |
| **Microvascular invasion (yes vs no)** | 11.791 | 0.001 | 0.934-2.148 | 0.101 |
| **Tumor capsule (none vs yes)** | 5.387 | 0.020 | 0.988-2.103 | 0.058 |
| **AFP(ng/ml) (≤400 vs >400)** | 0.248 | 0.594 | - | n.a. |
| **TNM stage** **(III-IV vs I-II)** | 2.501 | 0.114 | - | n.a. |
| **Tumor differentiation (III-IV vs I-II)** | 2.311 | 0.128 | - | n.a. |
| **Sex (male vs female)** | 2.151 | 0.142 | - | n.a. |
| **Liver cirrhosis (yes vs none)** | 2.974 | 0.085 | - | n.a. |
| **ALT (U/L) (≤75 vs >75)** | 0.196 | 0.658 | - | n.a. |
| **Age (years) (≤53vs >53)** | 0.886 | 0.347 | - | n.a. |
| **HBsAg (positive vs negative)** | 0.103 | 0.748 | - | n.a. |

**n.a., not applicable.**
