# Supplementary material for: CircMEMO1 modulates the promoter methylation and expression of TCF21 to regulate hepatocellular carcinoma progression and sorafenib treatment sensitivity
Source: Mol Cancer. 2021 May 13;20:75. doi: 10.1186/s12943-021-01361-3 (PMC8117652; doi:10.1186/s12943-021-01361-3)
Supplement: Supplementary file 7 — Additional file 7: Table S4 Univariate and Multivariate Analysis of Prognostic Factors of OS [file 12943_2021_1361_MOESM7_ESM.docx]

**TABLE S4 Univariate and Multivariate Analysis of Prognostic Factors of OS**

| **Variable** | **Univariate** | | **Multivariate** | |
| --- | --- | --- | --- | --- |
|  | χ2 | p value | HR(95%Cl) | p value |
| **TET1 expression (positive vs negative)** | 9.092 | 0.003 | 0.402-0.9754 | 0.038 |
| **Tumor diameter (cm) (>5 vs ≤5)** | 11.641 | 0.001 | 1.212-2.901 | 0.005 |
| **Tumor numble (≥2 vs 1)** | 3.967 | 0.046 | 0.712-2.127 | 0.458 |
| **Microvascular invasion (yes vs no)** | 14.527 | ＜0.001 | 1.122-2.855 | 0.015 |
| **Tumor capsule (none vs yes)** | 1.311 | 0.252 | - | n.a. |
| **AFP(ng/ml) (≤400 vs >400)** | 1.950 | 0.163 | - | n.a. |
| **TNM stage** **(III-IV vs I-II)** | 6.509 | 0.011 | - | n.a. |
| **Tumor differentiation (III-IV vs I-II)** | 2.835 | 0.092 | - | n.a. |
| **Sex(male vs female)** | 2.711 | 0.100 | - | n.a. |
| **Liver cirrhosis (positive vs negative)** | 0.270 | 0.603 | - | n.a. |
| **ALT (U/L) (≤75 vs >75)** | 0.600 | 0.439 | - | n.a. |
| **Age (years) (≤53vs >53)** | 1.323 | 0.250 | - | n.a. |
| **HBsAg (positive vs negative)** | 1.016 | 0.314 | - | n.a. |

**n.a., not applicable.**
