# Supplementary material for: CircMEMO1 modulates the promoter methylation and expression of TCF21 to regulate hepatocellular carcinoma progression and sorafenib treatment sensitivity
Source: Mol Cancer. 2021 May 13;20:75. doi: 10.1186/s12943-021-01361-3 (PMC8117652; doi:10.1186/s12943-021-01361-3)
Supplement: Supplementary file 8 — Additional file 8. Materials and Methods. [file 12943_2021_1361_MOESM8_ESM.docx]

**Materials and Methods**

**Cell Culture, Transfection, and RNA Extraction**

The human HCC cell lines Huh-7, PLC/PRF/5, Hep3B, MHCC97H, and HCCLM3 were routinely maintained in our laboratory. Transient transfection was performed using Lipofectamine 2000 (Invitrogen), and miRNAs were obtained using the mirVana™ miRNA Isolation Kit (Life Technologies) according to the manufacturer’s instructions.

**Clinical Specimens and Patient Follow-up**

In total, 209 specimens were randomly collected from consecutive patients with HCC who underwent curative resection at the Liver Cancer Institute of Fudan University (Shanghai, China). Fresh human HCC and adjacent non-tumour liver tissue samples were blindly collected from the cohort. Informed consent was obtained from each patient, and ethical approval was granted by the Ethics Committee of Zhongshan Hospital, Fudan University (Y2016-025). The follow-up procedures were described in detail in our previous studies (2,3,17).

**Plasmid Construction**

The lentiviral vectors pGMLV-SC5-shmiR-106b and pGMLV-MA2- pri-miR-106b and negative sequences were purchased from Gene Meditech (China). All plasmids were transfected into HEK293T cells using the HG transgene reagent according to the manufacturer’s instructions. After culturing for 48 h, the lentiviral particles in the supernatant were harvested and filtered by centrifugation at 500×g for 10 min. Human HCC cells were then transduced with the appropriate lentivirus. The plasmid pGMLV-SC5/pGMLV-MA2 expresses eGFP, and fluorescence was visualized to estimate the overall transfection efficiency. To select stably transduced cells, samples were resuspended and cultured with puromycin (2 μg/mL) for 2 weeks; quantitative reverse transcription-polymerase chain reaction (qRT-PCR) was then performed to determine the level of miR-106b-5p.

cDNA templates of TET family genes were cloned into the pPB-CAG vector and transfected into HCC cells using Lipofectamine 2000 (Invitrogen) according to the manufacturer’s instructions. Stably transfected clones were selected and validated by qRT-PCR and immunoblotting.

**miRNA Target Prediction**

The TargetScan (http://www.targetscan.org), miRBase (http://www.mirbase.org), miRanda (www.microrna.org), PicTar (http://pictar.mdc-berlin.de) and miRWalk (http://www.umm.uni-heidelberg.de/ apps/zmf/mirwalk) algorithms were used to screen for targets of miRNAs. The related functions of the evaluated miRNAs and their target genes were also examined.

**qRT-PCR**

miRNAs were extracted from cultured cells or fresh-frozen HCC tissue samples with the mirVana™ miRNA Isolation Kit (Life Technologies). Real-time PCR was performed in triplicate with the SYBR Green PCR method using an All-in-One miRNA *q*RT-PCR detection kit (GeneCopoeia Inc.). The forward primers and a common reverse primer were purchased from GeneCopoeia. All miRNA levels were normalized to the U6 small nuclear RNA level; mRNA levels were adjusted using β-actin as the reference. Relative expression was analysed by the comparative cycle threshold (Ct) method according to the equation 2-ΔCt [ΔCt = Ct- Ct (U6/β-actin)]. All experiments were performed in triplicate.

**Quantitative Analysis of 5hmC Levels Using Dot Blot Analysis**

Genomic DNA was denatured with 0.4 M NaOH and 10 mM ethylenediaminetetraacetic acid (EDTA) at 95°C for 10 min and then neutralized by adding an equal volume of cold 2 M ammonium acetate (pH 7.0). The DNA was then spotted on nitrocellulose membranes. After ultraviolet cross-linking, the membranes were blocked for 1 h with 5% bovine serum albumin (BSA) in Tris-buffered saline (TBS)+0.1% Tween 20 (TBST), followed by incubation with anti-5-hmC antibodies (1:10,000, Active Motif, catalogue No. 39769) overnight at 4°C; they were then subjected to western blotting detection.

**Immunofluorescence Assay and Western Blot Analysis**

An immunofluorescence assay and Western blot analysis were performed as described previously (17, 18). For 5-mC and 5hmC staining, samples were placed in 2 N HCl for 30 min, rinsed in distilled water, and placed in 100 mM Tris-HCl (pH 8.5) for 10 min before being blocked with PBS containing 5% BSA.

**Luciferase Assay**

The 3’-untranslated region (UTR) of human TET1/2 was amplified by PCR and cloned into the pGL3 vector to generate pGL3-TET1/2-3’UTR. This construct (2 ng) was cotransfected with 2 ng of pRL-TK plasmid. Luciferase activity was measured and normalized 48 h after transfection. Alternatively, cells were cotransfected with 200 ng of luciferase plasmids, 2 ng of pRL-TK and 20 nM miRNA or miRNA inhibitor.

**Immunocytochemistry**

Immunocytochemistry was performed as described previously (2). For 5-hmC staining, slides were placed in 2 N HCl for 30 min, rinsed in distilled water, and placed in 100 mM Tris-HCl (pH 8.5) for 10 min before being blocked with 5% normal goat serum at room temperature.

**In Situ Hybridization**

Tissue microarray (TMA) slides or 4-μm-thick formalin-fixed paraffin-embedded (FFPE) samples were incubated at 60°C for 1 h, deparaffinized in xylene, and rehydrated with graded alcohol washes. The slides were then washed three times with RNase-free PBS, digested with 8 mg/mL pepsin at 3°C for 10 min, washed, and dehydrated in graded alcohol washes. The slides were hybridized overnight at 40°C with 50 nM locked nucleic acid (LNA)-modified DIG-labelled probes for miR-106b. After stringency washes (5×, 1×, and 0.2× saline-sodium citrate (SSC)), the slides were placed in blocking buffer for 30 min at room temperature, followed by overnight incubation at 4°C in an alkaline phosphatase-conjugated anti-DIG Fab fragment solution. The antibody signal was developed with a 5-bromo-4-chloro-3-indolyl phosphate (BCIP)/nitro blue tetrazolium (NBT) substrate (Roche, Mannheim, Germany), and nuclei were stained with Nuclear Fast Red.

**Migration and Invasion Assays**

Twenty-four-well Boyden chambers with an 8-μm-pore polycarbonate membrane (Corning, Cambridge, MA) were used to analyse tumour cell migration. For invasion assays, the membrane was coated with Matrigel to form a matrix barrier.

**In Vivo Metastasis Assays**

For in vivo metastasis assays, nude mice were injected with 5×10^6^ cells suspended in 40 μL of serum-free DMEM/Matrigel (1:1). After 6 weeks, the mice were sacrificed, and the lungs and liver were harvested, fixed with phosphate-buffered neutral formalin and prepared for standard histological examination.

**Statistical Analysis**

Statistical analysis was performed with SPSS 19.0 software (SPSS). All tests were two tailed, and *p*<0.05 was considered statistically significant.
